# RETRACTED: El-Gowily et al. Tioconazole and Chloroquine Act Synergistically to Combat Doxorubicin-Induced Toxicity via Inactivation of PI3K/AKT/mTOR Signaling Mediated ROS-Dependent Apoptosis and Autophagic Flux Inhibition in MCF-7 Breast Cancer Cells. *Pharmaceuticals* 2021, *14*, 254

**DOI:** 10.3390/ph18030338

**Published:** 2025-02-27

**Authors:** Afnan H. El-Gowily, Samah A. Loutfy, Ehab M. M. Ali, Tarek M. Mohamed, Mohammed A. Mansour

**Affiliations:** 1 Biochemistry Division, Department of Chemistry, Faculty of Science, Tanta University, Tanta 31527, Egypt; 2 Virology & Immunology Unit, Cancer Biology Department, National Cancer Institute, Cairo University, Cairo, Egypt; samah.loutfy@bue.edu.eg; 3 Nanotechnology Research Center, British University, Cairo, Egypt; 4 Department of Biochemistry, Faculty of Science, King Abdulaziz University, Jeddah, Saudi Arabia; emali@kau.edu.sa; 5 Chemistry Department, Faculty of Science, Tanta University, Tanta 31527, Egypt; 6 Division of Human Sciences, School of Applied Sciences, London South Bank University, London SE1 0AA, UK

The journal retracts the article titled “Tioconazole and Chloroquine Act Synergistically to Combat Doxorubicin-Induced Toxicity via Inactivation of PI3K/AKT/mTOR Signaling Mediated ROS-Dependent Apoptosis and Autophagic Flux Inhibition in MCF-7 Breast Cancer Cells.” [1], cited above.

Following the article’s publication, concerns were brought to the attention of the Editorial Office regarding the reliability of the figures presented in this article [1].

Adhering to our complaints procedure, the investigation was conducted by the Editorial Office and Editorial Board. During this process, the authors were unable to satisfactorily explain the similarities between Figure 4(I-A) and Supplementary Figure S5(C II), nor could they provide the raw materials for the Editorial Board evaluation. As a result, the Editorial Board has lost confidence in the reliability of the findings and decided to retract this publication [1], as per MDPI’s retraction policy (https://www.mdpi.com/ethics#_bookmark30, accessed on 6 January 2025).

This retraction was approved by the Editor-in-Chief of *Pharmaceuticals*. 

The authors disagree with this decision. 
